# Crystal structure and Hirshfeld surface analysis of (*E*)-3-benzyl­idene-4-oxo­penta­noic acid

**DOI:** 10.1107/S2056989022004789

**Published:** 2022-05-13

**Authors:** Hamza Assila, Younes Zaoui, Issam Ameziane El Hassani, Samira El Ghammarti, Joel T. Mague, Jamal Taoufik, Youssef Ramli, Mhammed Ansar

**Affiliations:** a Laboratory of Medicinal Chemistry, Drug Sciences Research Center, Faculty of Medicine and Pharmacy, Mohammed V University in Rabat, Rabat, Morocco; b Medical and Clinical Biology Department Faculty of Medicine and Pharmacy, Mohammed V University in Rabat, Rabat, Morocco; c Department of Chemistry, Tulane University, New Orleans, LA 70118, USA; Katholieke Universiteit Leuven, Belgium

**Keywords:** crystal structure, hydrogen bond, carb­oxy­lic acid, Hirshfeld surface

## Abstract

The asymmetric unit of the title mol­ecule, C_12_H_12_O_3_, contains two independent mol­ecules having opposite conformations and each forming self-dimers through complementary O—H⋯O hydrogen bonds. These dimers are linked by weak C—H⋯π inter­actions and C—H⋯O hydrogen bonds into a three-dimensional structure in which one can discern layers parallel to the *bc* plane. A Hirshfeld surface analysis of the inter­molecular inter­actions is included.

## Abstract 2

The asymmetric unit contains two independent mol­ecules having opposite conformations and each forming self-dimers through complementary O—H⋯O hydrogen bonds. These dimers are linked by weak C—H⋯π inter­actions involving the phenyl ring and the olefinic double bond into zigzag chains extending along the *c*-axis direction. The chains are linked by C—H⋯O hydrogen bonds to form the full three-dimensional structure in which one can discern layers parallel to the *bc* plane.

## 1. Chemical context

Levulinic acid has various derivatives, some of which have a wide range of pharmacological activities. Photodynamic therapy in gastroenterology (Mordon *et al.*, 2005 ▸) and cancer treatment for the detection of tumor tissue (Manzo, 2012 ▸) are some of the pharmacological applications. These derivatives are also the main compounds used in the synthesis of some pyridazinone derivatives (Boukharsa *et al.*, 2016*a* ▸,*b* ▸; Zaoui *et al.*, 2019 ▸, 2021 ▸). In our research, great attention has been given to the development of diversely functionalized heterocycles (Guerrab *et al.*, 2020 ▸, 2021 ▸; Abad *et al.* 2021 ▸; Missioui *et al.*, 2021 ▸, 2022*a* ▸,*b* ▸). Given the wide range of therapeutic applications for such compounds, and in continuation of our research efforts, we report the synthesis, mol­ecular and crystal structure and a Hirshfeld surface analysis of the title compound (see Scheme[Chem scheme1]). 
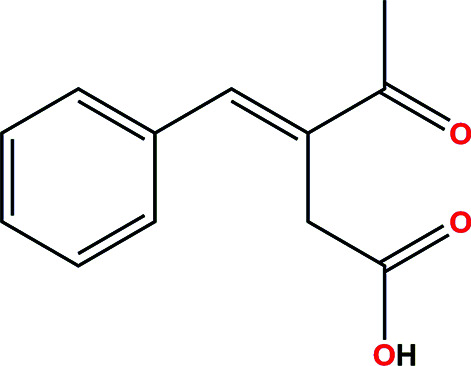


## 2. Structural commentary

**Figure 1:**
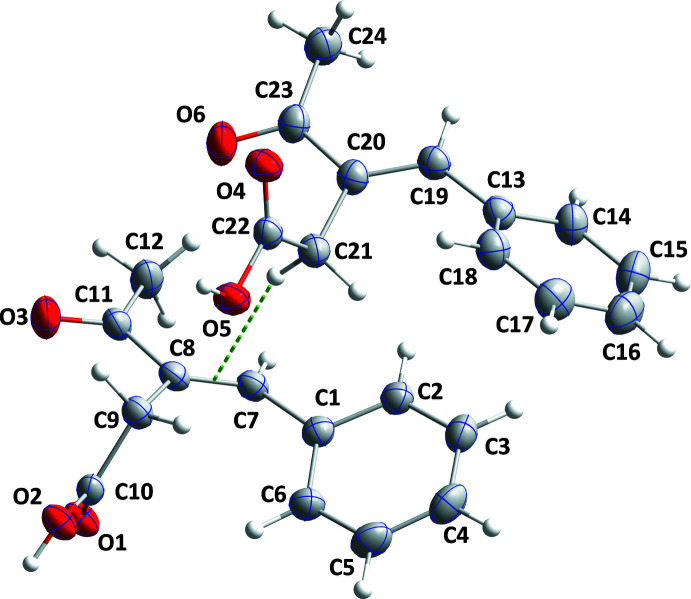
The asymmetric unit of the title compound showing the atom-labelling scheme and 50% probability displacement ellipsoids. The C—H⋯π inter­action is depicted by a dashed line.

**Figure 2:**
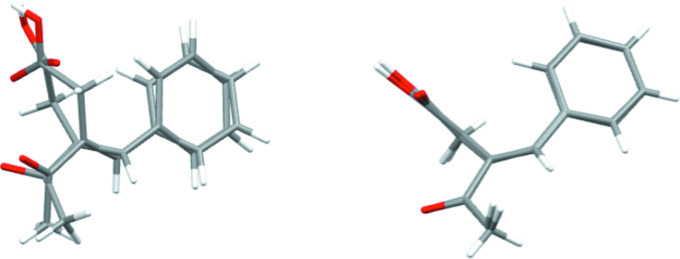
Overlay of the two independent mol­ecules as found (left) and with the second one inverted (right).

The asymmetric unit consists of two independent mol­ecules (Fig. 1 ▸) having opposite configurations, as shown in Fig. 2 ▸, where inverting the mol­ecule containing atom O4 allows almost complete overlap between the two independent portions of the asymmetric unit [r.m.s. deviations = 1.204 (no inversion) and 0.163 Å (inversion)]. They also differ in the dihedral angle between their planar parts. Thus, the C2—C1—C7—C8 torsion angle is −143.15 (14)°, while the C14—C13—C19—C20 torsion angle is 139.55 (15)°. The dihedral angle between the mean plane of the C1–C6 phenyl ring and that defined by atoms C7–C9/C11 is 36.54 (5)° in one mol­ecule, while that between the C13–C18 ring and the plane defined by atoms C19–C21/C23 in the other mol­ecule is 41.67 (6)°. In the first mol­ecule, the dihedral angle between the best planes through C7–C9/C11 and C9/C10/O1/O2 is 81.96 (5)°, while that between the C19–C21/C23 and C21/C22/O4/O5 planes in the second mol­ecule is 75.53 (6)°. Finally, the dihedral angle between the mean C8/C11/C12/O3 and C7–C9/C11 planes in the first mol­ecule is 2.88 (12)°, while that between the mean C20/C23/C24/O6 and C19–C21/C23 planes in the second mol­ecule is 5.22 (3)°. All bond lengths and angles are as expected.

## 3. Supra­molecular features

**Figure 3:**
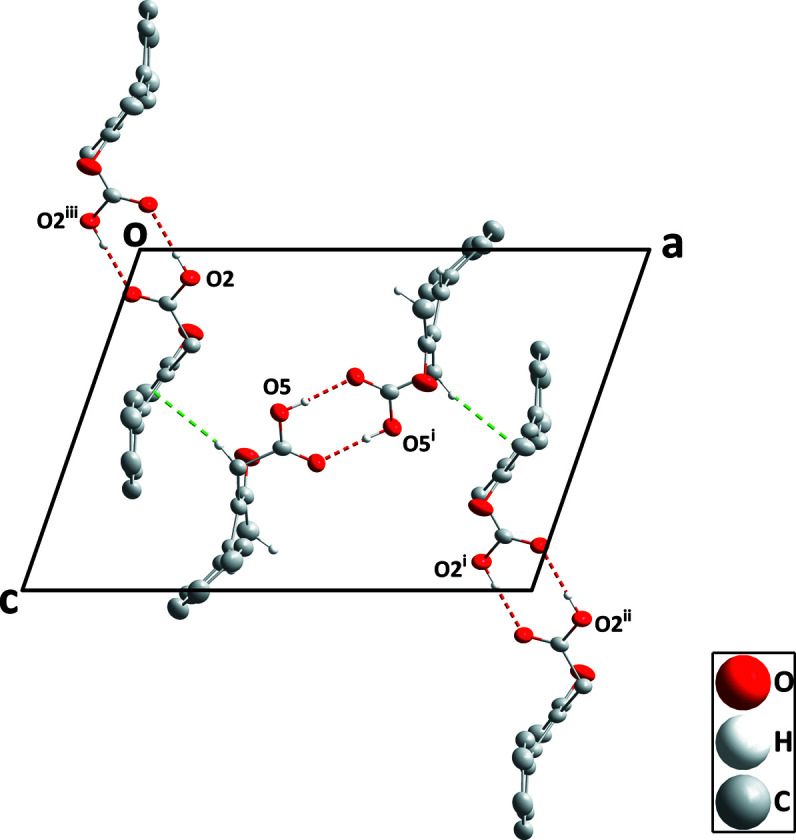
Detail of the inter­actions between hydrogen-bonded dimers viewed along the *b*-axis direction. The O—H⋯O hydrogen bonds and the C—H⋯π inter­actions are depicted, respectively, by red and green dashed lines. Non-inter­acting H atoms have been omitted for clarity. [Symmetry codes: (i) −*x* + 1, −*y* + 1, −*z* + 1; (ii) *x* + 1, *y*, *z* + 1; (iii) −*x*, −*y* + 1, −*z*.]

**Figure 4:**
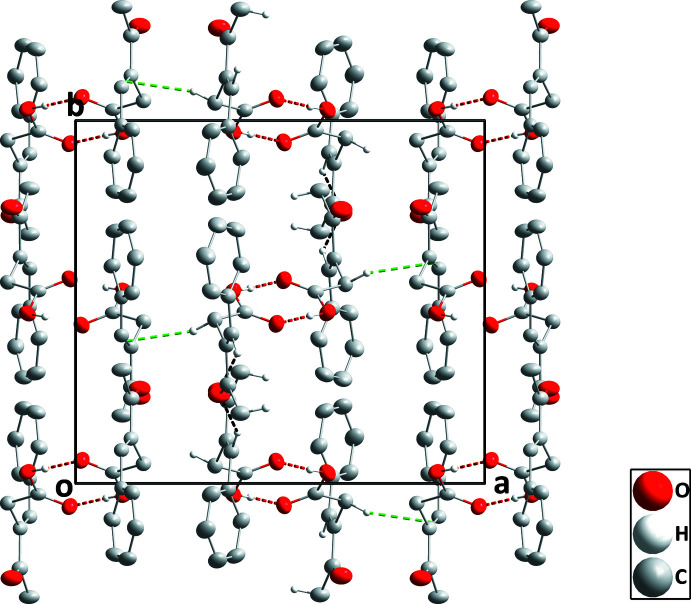
Packing viewed along the *c*-axis direction with O—H⋯O and C—H⋯O hydrogen bonds depicted, respectively, by red and black dashed lines. The C—H⋯π inter­actions are depicted by green dashed lines and non-inter­acting H atoms have been omitted for clarity.

In the crystal, each independent mol­ecule forms a centrosymmetric self-dimer with the dimers connected by a C—H⋯π inter­action between C21—H21*A* and the C7=C8 olefinic bond [H21*A*⋯*Cg* = 2.60 Å, C21⋯*Cg* = 3.547 (2) Å and C21—H21*A*⋯*Cg* = 161°; *Cg* is the centroid of C7=C8; see Table 1 ▸ and Fig. 3 ▸]. The unit shown in Fig. 3 ▸ is linked to others through weak C19—H19⋯O6 hydrogen bonds (Table 1 ▸) to form a three-dimensional network structure. Although these inter­molecular inter­actions propagate in three dimensions, one can discern layers constructed by the hydrogen-bond inter­actions which are connected by the C—H⋯π inter­actions. These layers are parallel to the *bc* plane (Fig. 4 ▸).

## 4. Database survey

A search of the Cambridge Structural Database updated to November 2021 (Groom *et al.*, 2016 ▸) with a search fragment consisting of the title mol­ecule with H2*A* and H7, as well as all H atoms on the phenyl ring deleted, found mainly bicyclic mol­ecules not closely related to the title mol­ecule. Using the above search fragment but with H7 now present, one hit, namely, 3-(4-methyl­benzyl­idene)-4-oxo­penta­noic acid (CSD refcode UCOXOC; Boukharsa *et al.*, 2016*a* ▸,*b* ▸) was obtained (also found in the previous search). This structure also contains two independent mol­ecules (*A* and *B*) which form *A–A* and *B–B* hydrogen-bonded inversion dimers, as seen in the present structure. The packing in UCOXOC appears to generate also a layer structure, but no mention is made of additional inter­molecular inter­actions.

## 5. Hirshfeld surface analysis

**Figure 5:**
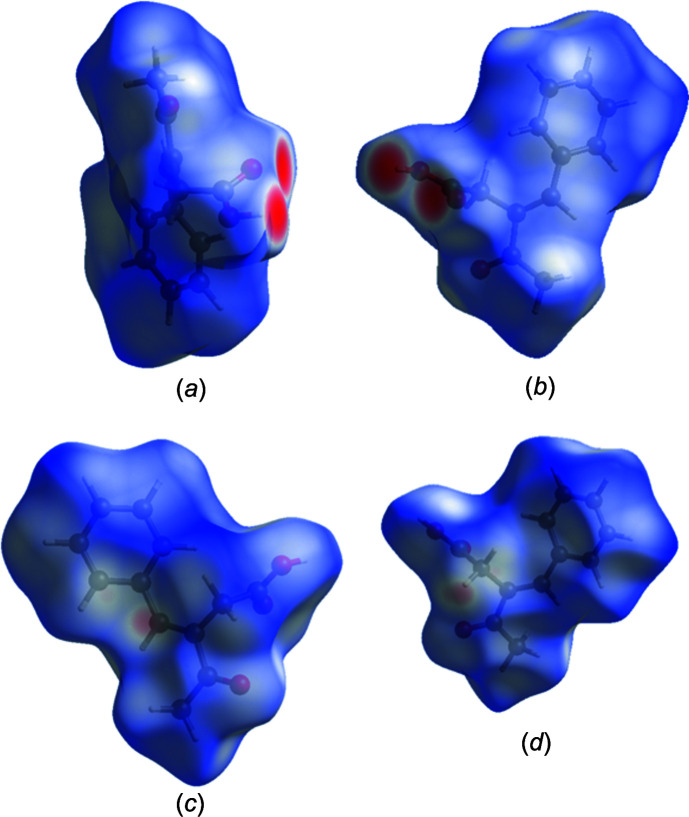
The Hirshfeld surface plots for the title mol­ecule: (*a*) *d*_norm_ for the O1-containing mol­ecule (front side); (*b*) *d*_norm_ for the O4-containing mol­ecule (front side); (*c*) *d*_norm_ for the O1-containing mol­ecule (back side); (*d*) *d*_norm_ for the O4-containing mol­ecule (back side).

**Figure 6:**
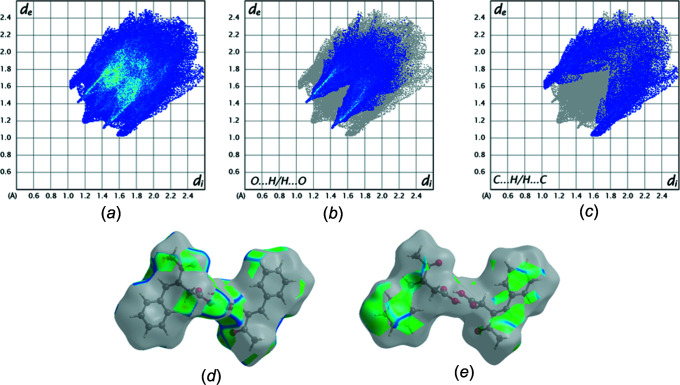
Fingerprint plots for the title mol­ecule: (*a*) all inter­actions; (*b*) O⋯H/H⋯O; (*c*) C⋯H/H⋯C; (*d*) fragment of the surface involved in O⋯H/H⋯O inter­actions; (*e*) fragment of the surface involved in C⋯H/H⋯C inter­actions.

The Hirshfeld surface analysis was performed with *CrystalExplorer* (Version 21.5; Spackman *et al.*, 2021 ▸); the details of the pictorial output are described in a recent publication (Tan *et al.*, 2019 ▸). Fig. 5 ▸ shows two views of the *d*_norm_ surfaces for the two components of the asymmetric unit plotted over the limits from −0.1211 to 1.4747 a.u. The O—H⋯O hydrogen bonds with which each mol­ecule forms its self-dimer are indicated by the bright red spots in Figs. 5 ▸(*a*) and 5(*b*), respectively. The weak inter­molecular C—H⋯π inter­action with the olefinic double bond appears in Fig. 5 ▸(*c*) as the lighter red spot in the centre of the left side of the drawing, showing the acceptor site, and in a similar location in Fig. 5 ▸(*d*), showing the donor site. Fig. 6 ▸ presents the two-dimensional fingerprint plots involving all inter­molecular inter­actions [Fig. 6 ▸(*a*)] and delineated into O⋯H/H⋯O [Fig. 6 ▸(*b*)] and C⋯H/H⋯C [Fig. 6 ▸(*c*)] inter­actions. Figs. 6 ▸(*d*) and 6(*e*) show the fractions of the overall surface corresponding, respectively, to the two above inter­actions (28.8% for the fomer and 18.2% for the latter). For completeness, the H⋯H inter­actions constitute 48.4% of the surface.

## 6. Synthesis and crystallization

A mixture of benzaldehyde (0.01 mol) and levulinic acid (0.02 mol) in a solution of acetic acid (50 ml) was saturated with dry hydrogen chloride gas for 2 h. The mixture was stirred at room temperature for 24 h. The resulting product was extracted and washed with chloro­form. The crude compound was crystallized from acetone to give small colourless crystals (yield: 59%; m.p 398–400 K). IR (KBr, ν (cm^−1^)): 1692 (C=O ketone), 1755 (C=O acid); ^1^H NMR [300 MHz DMSO-*d*_6_, δ(ppm)]: δ 2.42 (*s*, 3H, CH_3_), 3.74 (*s*, 2H, CH_2_), 7.27–7.75 (*m*, 5H, phen­yl), 7.98 (*s*, 1H, CH=C), 12.21 (*s*, 1H, OH); ^13^C NMR [300 MHz DMSO-*d*_6_, δ(ppm)]: δ 26.10, 32.83, 128.01, 131,09, 131.52, 133.79, 137.32, 137.43, 171.78, 192.72; MS (ESI+): *m*/*z* = 205.88 [*M* + H]^+^

## 7. Refinement

Crystal data, data collection and structure refinement details are summarized in Table 2 ▸. H atoms attached to carbon were placed in idealized positions and included as riding contributions with isotropic displacement parameters 1.2–1.5 times those of the attached atoms. H atoms attached to oxygen were placed in locations derived from a difference map and refined with a DFIX 0.84 0.01 instruction.

## Supplementary Material

Crystal structure: contains datablock(s) global, I. DOI: 10.1107/S2056989022004789/vm2263sup1.cif

Structure factors: contains datablock(s) I. DOI: 10.1107/S2056989022004789/vm2263Isup2.hkl

CCDC reference: 2170436

Additional supporting information:  crystallographic information; 3D view; checkCIF report

## Figures and Tables

**Table 1:** Hydrogen-bond geometry (Å, °)

*D*—H⋯*A*	*D*—H	H⋯*A*	*D*⋯*A*	*D*—H⋯*A*
O2—H2*A*⋯O1^i^	0.84 (1)	1.78 (1)	2.6226 (13)	178 (2)
O5—H5*A*⋯O4^ii^	0.86 (1)	1.74 (1)	2.6000 (13)	176 (2)
C19—H19⋯O6^iii^	0.95	2.60	3.447 (1)	148

Symmetry codes: (i) 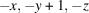 ; (ii) 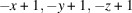 ; (iii) 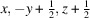 .

**Table 2:** Experimental details

Crystal data	
Chemical formula	C_12_H_12_O_3_
*M*_r_	204.22
Crystal system, space group	Monoclinic, *P*2_1_/*c*
Temperature (K)	125
*a*, *b*, *c* (Å)	15.5987 (3), 13.0782 (3), 11.0396 (2)
β (°)	109.063 (1)
*V* (Å^3^)	2128.60 (8)
*Z*	8
Radiation type	Cu *K*α
μ (mm^−1^)	0.75
Crystal size (mm)	0.35 × 0.18 × 0.07

Data collection	
Diffractometer	Bruker D8 VENTURE PHOTON 3 CPAD
Absorption correction	Multi-scan (*SADABS*; Krause *et al.*, 2015 ▸)
*T*_min_, *T*_max_	0.85, 0.95
No. of measured, independent and observed [*I* > 2σ(*I*)] reflections	36991, 3894, 3477
*R*_int_	0.042
(sin θ/λ)_max_ (Å^−1^)	0.603

Refinement	
*R*[*F* ^2^ > 2σ(*F* ^2^)], *wR*(*F* ^2^), *S*	0.037, 0.098, 1.04
No. of reflections	3894
No. of parameters	281
No. of restraints	2
H-atom treatment	H atoms treated by a mixture of independent and constrained refinement
Δρ_max_, Δρ_min_ (e Å^−3^)	0.26, −0.16

Computer programs: *APEX4* and *SAINT* (Bruker, 2021 ▸), *SHELXT* (Sheldrick, 2015*a* ▸), *SHELXL2018* (Sheldrick, 2015*b* ▸), *DIAMOND* (Brandenburg & Putz, 2012 ▸) and *SHELXTL* (Bruker, 2021 ▸).

## supplementary crystallographic information

### Crystal data

**Table:**

C_12_H_12_O_3_	*F*(000) = 864
*M**_r_* = 204.22	*D*_x_ = 1.274 Mg m^−^^3^
Monoclinic, *P*2_1_/*c*	Cu *K*α radiation, λ = 1.54178 Å
*a* = 15.5987 (3) Å	Cell parameters from 9897 reflections
*b* = 13.0782 (3) Å	θ = 3.0–68.3°
*c* = 11.0396 (2) Å	µ = 0.75 mm^−^^1^
β = 109.063 (1)°	*T* = 125 K
*V* = 2128.60 (8) Å^3^	Plate, colourless
*Z* = 8	0.35 × 0.18 × 0.07 mm

### Data collection

**Table:**

Bruker D8 VENTURE PHOTON 3 CPAD diffractometer	3894 independent reflections
Radiation source: INCOATEC IµS micro-focus source	3477 reflections with *I* > 2σ(*I*)
Mirror monochromator	*R*_int_ = 0.042
Detector resolution: 7.3910 pixels mm^-1^	θ_max_ = 68.3°, θ_min_ = 3.0°
ω and φ scans	*h* = −18→18
Absorption correction: multi-scan (*SADABS*; Krause *et al.*, 2015)	*k* = −15→15
*T*_min_ = 0.85, *T*_max_ = 0.95	*l* = −13→13
36991 measured reflections	

### Refinement

**Table:**

Refinement on *F*^2^	Primary atom site location: dual
Least-squares matrix: full	Secondary atom site location: difference Fourier map
*R*[*F*^2^ > 2σ(*F*^2^)] = 0.037	Hydrogen site location: mixed
*wR*(*F*^2^) = 0.098	H atoms treated by a mixture of independent and constrained refinement
*S* = 1.04	*w* = 1/[σ^2^(*F*_o_^2^) + (0.0449*P*)^2^ + 0.7261*P*] where *P* = (*F*_o_^2^ + 2*F*_c_^2^)/3
3894 reflections	(Δ/σ)_max_ < 0.001
281 parameters	Δρ_max_ = 0.26 e Å^−^^3^
2 restraints	Δρ_min_ = −0.15 e Å^−^^3^

### Special details

**Table:**

Experimental. The diffraction data were obtained from 13 sets of frames, each of width 0.5° in ω or φ, collected with scan parameters determined by the "strategy" routine in *APEX3*. The scan time varied between 4 and 10 sec/frame, increasing with increasing θ.
Geometry. All esds (except the esd in the dihedral angle between two l.s. planes) are estimated using the full covariance matrix. The cell esds are taken into account individually in the estimation of esds in distances, angles and torsion angles; correlations between esds in cell parameters are only used when they are defined by crystal symmetry. An approximate (isotropic) treatment of cell esds is used for estimating esds involving l.s. planes.
Refinement. Refinement of F^2^ against ALL reflections. The weighted R-factor wR and goodness of fit S are based on F^2^, conventional R-factors R are based on F, with F set to zero for negative F^2^. The threshold expression of F^2^ > 2sigma(F^2^) is used only for calculating R-factors(gt) etc. and is not relevant to the choice of reflections for refinement. R-factors based on F^2^ are statistically about twice as large as those based on F, and R- factors based on ALL data will be even larger. H-atoms attached to carbon were placed in calculated positions (C—H = 0.95 - 0.99 Å) and included as riding contributions with isotropic displacement parameters 1.2 - 1.5 times those of the attached atoms. Those attached to oxygen were placed in locations derived from a difference map and refined with a DFIX 0.84 0.01 instruction.

### Fractional atomic coordinates and isotropic or equivalent isotropic displacement parameters (Å^2^)

**Table:**

	*x*	*y*	*z*	*U*_iso_*/*U*_eq_	
O1	0.01466 (6)	0.43636 (7)	0.13158 (8)	0.0335 (2)	
O2	0.11775 (6)	0.53207 (7)	0.08370 (9)	0.0330 (2)	
H2A	0.0747 (10)	0.5405 (15)	0.0145 (12)	0.059 (6)*	
O3	0.15617 (8)	0.23912 (8)	0.24312 (9)	0.0428 (3)	
C1	0.11190 (8)	0.51281 (10)	0.51675 (12)	0.0287 (3)	
C2	0.13778 (9)	0.52531 (10)	0.64944 (13)	0.0319 (3)	
H2	0.149625	0.466767	0.703342	0.038*	
C3	0.14645 (10)	0.62214 (11)	0.70370 (14)	0.0381 (3)	
H3	0.165783	0.629690	0.794220	0.046*	
C4	0.12690 (10)	0.70747 (11)	0.62572 (16)	0.0425 (4)	
H4	0.134096	0.773842	0.662664	0.051*	
C5	0.09682 (11)	0.69635 (11)	0.49382 (16)	0.0425 (4)	
H5	0.081391	0.755016	0.440322	0.051*	
C6	0.08917 (9)	0.59974 (11)	0.43962 (14)	0.0352 (3)	
H6	0.068235	0.592658	0.349008	0.042*	
C7	0.11215 (9)	0.40800 (10)	0.46866 (12)	0.0283 (3)	
H7	0.093428	0.356028	0.514695	0.034*	
C8	0.13551 (8)	0.37633 (10)	0.36773 (11)	0.0277 (3)	
C9	0.16595 (9)	0.44565 (10)	0.28089 (12)	0.0296 (3)	
H9A	0.189225	0.510133	0.326857	0.036*	
H9B	0.216439	0.412547	0.259955	0.036*	
C10	0.09137 (9)	0.47015 (9)	0.15870 (11)	0.0263 (3)	
C11	0.13621 (9)	0.26565 (10)	0.33666 (12)	0.0308 (3)	
C12	0.11327 (11)	0.18699 (11)	0.42061 (14)	0.0390 (3)	
H12A	0.051375	0.198520	0.421374	0.059*	
H12B	0.155908	0.192707	0.508058	0.059*	
H12C	0.117594	0.118474	0.387117	0.059*	
O4	0.49089 (6)	0.43991 (8)	0.62632 (9)	0.0363 (2)	
O5	0.38438 (7)	0.52938 (8)	0.47860 (9)	0.0362 (2)	
H5A	0.4271 (11)	0.5378 (16)	0.4462 (19)	0.068 (6)*	
O6	0.35094 (8)	0.24526 (8)	0.60897 (9)	0.0465 (3)	
C13	0.37323 (9)	0.51643 (11)	0.91953 (12)	0.0321 (3)	
C14	0.33171 (10)	0.52973 (12)	1.01313 (14)	0.0390 (3)	
H14	0.312493	0.471674	1.049168	0.047*	
C15	0.31836 (11)	0.62700 (13)	1.05381 (16)	0.0465 (4)	
H15	0.289284	0.635179	1.116582	0.056*	
C16	0.34705 (11)	0.71187 (12)	1.00363 (16)	0.0484 (4)	
H16	0.336386	0.778415	1.030129	0.058*	
C17	0.39153 (11)	0.69983 (12)	0.91436 (15)	0.0446 (4)	
H17	0.412634	0.758112	0.881168	0.053*	
C18	0.40518 (10)	0.60291 (11)	0.87360 (14)	0.0376 (3)	
H18	0.436686	0.595157	0.813635	0.045*	
C19	0.38059 (9)	0.41173 (10)	0.87494 (13)	0.0313 (3)	
H19	0.395855	0.359823	0.938630	0.038*	
C20	0.36805 (9)	0.38144 (10)	0.75365 (12)	0.0302 (3)	
C21	0.34049 (9)	0.45106 (11)	0.63929 (12)	0.0314 (3)	
H21A	0.288072	0.420238	0.572571	0.038*	
H21B	0.320109	0.516898	0.664775	0.038*	
C22	0.41342 (9)	0.47210 (10)	0.58225 (12)	0.0287 (3)	
C23	0.37236 (9)	0.27147 (11)	0.72132 (13)	0.0339 (3)	
C24	0.40272 (11)	0.19335 (11)	0.82582 (15)	0.0418 (3)	
H24A	0.399988	0.125163	0.787939	0.063*	
H24B	0.362870	0.195663	0.878428	0.063*	
H24C	0.465178	0.208109	0.879500	0.063*	

### Atomic displacement parameters (Å^2^)

**Table:**

	*U*^11^	*U*^22^	*U*^33^	*U*^12^	*U*^13^	*U*^23^
O1	0.0347 (5)	0.0383 (5)	0.0257 (5)	−0.0049 (4)	0.0075 (4)	0.0069 (4)
O2	0.0388 (5)	0.0329 (5)	0.0257 (5)	−0.0063 (4)	0.0082 (4)	0.0062 (4)
O3	0.0669 (7)	0.0353 (5)	0.0304 (5)	−0.0014 (5)	0.0219 (5)	−0.0038 (4)
C1	0.0276 (6)	0.0284 (7)	0.0310 (6)	0.0001 (5)	0.0108 (5)	0.0006 (5)
C2	0.0347 (7)	0.0313 (7)	0.0319 (7)	0.0028 (5)	0.0139 (5)	0.0011 (5)
C3	0.0397 (8)	0.0382 (8)	0.0382 (7)	0.0013 (6)	0.0154 (6)	−0.0083 (6)
C4	0.0470 (8)	0.0284 (7)	0.0564 (9)	−0.0016 (6)	0.0229 (7)	−0.0088 (7)
C5	0.0514 (9)	0.0283 (7)	0.0521 (9)	0.0054 (6)	0.0226 (7)	0.0065 (7)
C6	0.0395 (7)	0.0314 (7)	0.0348 (7)	0.0039 (6)	0.0123 (6)	0.0036 (6)
C7	0.0311 (6)	0.0269 (6)	0.0253 (6)	−0.0004 (5)	0.0070 (5)	0.0034 (5)
C8	0.0299 (6)	0.0280 (6)	0.0220 (6)	−0.0003 (5)	0.0040 (5)	0.0031 (5)
C9	0.0328 (7)	0.0298 (7)	0.0257 (6)	−0.0010 (5)	0.0088 (5)	0.0023 (5)
C10	0.0359 (7)	0.0216 (6)	0.0231 (6)	0.0003 (5)	0.0120 (5)	−0.0004 (5)
C11	0.0375 (7)	0.0303 (7)	0.0224 (6)	0.0000 (5)	0.0067 (5)	−0.0002 (5)
C12	0.0595 (9)	0.0266 (7)	0.0334 (7)	−0.0015 (6)	0.0185 (7)	−0.0001 (6)
O4	0.0337 (5)	0.0428 (6)	0.0339 (5)	0.0038 (4)	0.0131 (4)	0.0084 (4)
O5	0.0373 (5)	0.0402 (6)	0.0340 (5)	0.0066 (4)	0.0155 (4)	0.0103 (4)
O6	0.0692 (7)	0.0404 (6)	0.0334 (5)	−0.0001 (5)	0.0218 (5)	−0.0070 (5)
C13	0.0336 (7)	0.0330 (7)	0.0278 (6)	−0.0023 (5)	0.0074 (5)	−0.0018 (5)
C14	0.0483 (8)	0.0373 (8)	0.0347 (7)	−0.0060 (6)	0.0183 (6)	−0.0047 (6)
C15	0.0520 (9)	0.0456 (9)	0.0470 (9)	−0.0059 (7)	0.0232 (7)	−0.0146 (7)
C16	0.0508 (9)	0.0358 (8)	0.0566 (10)	−0.0040 (7)	0.0149 (8)	−0.0151 (7)
C17	0.0501 (9)	0.0326 (8)	0.0489 (9)	−0.0104 (6)	0.0133 (7)	−0.0038 (7)
C18	0.0409 (8)	0.0372 (8)	0.0359 (7)	−0.0069 (6)	0.0141 (6)	−0.0040 (6)
C19	0.0346 (7)	0.0309 (7)	0.0301 (7)	−0.0015 (5)	0.0128 (5)	0.0023 (5)
C20	0.0313 (7)	0.0310 (7)	0.0312 (7)	−0.0024 (5)	0.0141 (5)	0.0003 (5)
C21	0.0320 (7)	0.0342 (7)	0.0285 (6)	0.0003 (5)	0.0104 (5)	−0.0010 (5)
C22	0.0363 (7)	0.0249 (6)	0.0248 (6)	−0.0014 (5)	0.0098 (5)	−0.0016 (5)
C23	0.0377 (7)	0.0345 (7)	0.0334 (7)	−0.0033 (6)	0.0168 (6)	−0.0026 (6)
C24	0.0533 (9)	0.0314 (7)	0.0414 (8)	−0.0001 (6)	0.0164 (7)	0.0000 (6)

### Geometric parameters (Å, º)

**Table:**

O1—C10	1.2177 (15)	O4—C22	1.2207 (16)
O2—C10	1.3165 (15)	O5—C22	1.3180 (16)
O2—H2A	0.843 (9)	O5—H5A	0.860 (9)
O3—C11	1.2221 (16)	O6—C23	1.2234 (17)
C1—C6	1.3950 (19)	C13—C18	1.396 (2)
C1—C2	1.3961 (18)	C13—C14	1.3981 (19)
C1—C7	1.4704 (18)	C13—C19	1.4722 (19)
C2—C3	1.3885 (19)	C14—C15	1.387 (2)
C2—H2	0.9500	C14—H14	0.9500
C3—C4	1.381 (2)	C15—C16	1.379 (2)
C3—H3	0.9500	C15—H15	0.9500
C4—C5	1.384 (2)	C16—C17	1.387 (2)
C4—H4	0.9500	C16—H16	0.9500
C5—C6	1.386 (2)	C17—C18	1.385 (2)
C5—H5	0.9500	C17—H17	0.9500
C6—H6	0.9500	C18—H18	0.9500
C7—C8	1.3458 (18)	C19—C20	1.3479 (18)
C7—H7	0.9500	C19—H19	0.9500
C8—C11	1.4885 (18)	C20—C23	1.4887 (19)
C8—C9	1.5046 (17)	C20—C21	1.5011 (18)
C9—C10	1.5002 (17)	C21—C22	1.4949 (18)
C9—H9A	0.9900	C21—H21A	0.9900
C9—H9B	0.9900	C21—H21B	0.9900
C11—C12	1.5040 (18)	C23—C24	1.497 (2)
C12—H12A	0.9800	C24—H24A	0.9800
C12—H12B	0.9800	C24—H24B	0.9800
C12—H12C	0.9800	C24—H24C	0.9800
			
C10—O2—H2A	109.3 (13)	C22—O5—H5A	109.9 (14)
C6—C1—C2	118.28 (12)	C18—C13—C14	118.30 (13)
C6—C1—C7	124.71 (12)	C18—C13—C19	123.78 (12)
C2—C1—C7	117.00 (11)	C14—C13—C19	117.92 (12)
C3—C2—C1	120.89 (13)	C15—C14—C13	120.54 (14)
C3—C2—H2	119.6	C15—C14—H14	119.7
C1—C2—H2	119.6	C13—C14—H14	119.7
C4—C3—C2	119.83 (13)	C16—C15—C14	120.35 (14)
C4—C3—H3	120.1	C16—C15—H15	119.8
C2—C3—H3	120.1	C14—C15—H15	119.8
C3—C4—C5	120.05 (13)	C15—C16—C17	119.83 (14)
C3—C4—H4	120.0	C15—C16—H16	120.1
C5—C4—H4	120.0	C17—C16—H16	120.1
C4—C5—C6	120.11 (14)	C18—C17—C16	120.04 (14)
C4—C5—H5	119.9	C18—C17—H17	120.0
C6—C5—H5	119.9	C16—C17—H17	120.0
C5—C6—C1	120.69 (13)	C17—C18—C13	120.83 (13)
C5—C6—H6	119.7	C17—C18—H18	119.6
C1—C6—H6	119.7	C13—C18—H18	119.6
C8—C7—C1	128.26 (12)	C20—C19—C13	127.13 (13)
C8—C7—H7	115.9	C20—C19—H19	116.4
C1—C7—H7	115.9	C13—C19—H19	116.4
C7—C8—C11	120.96 (12)	C19—C20—C23	121.25 (12)
C7—C8—C9	124.70 (12)	C19—C20—C21	124.47 (12)
C11—C8—C9	114.30 (11)	C23—C20—C21	114.03 (11)
C10—C9—C8	112.85 (10)	C22—C21—C20	114.69 (11)
C10—C9—H9A	109.0	C22—C21—H21A	108.6
C8—C9—H9A	109.0	C20—C21—H21A	108.6
C10—C9—H9B	109.0	C22—C21—H21B	108.6
C8—C9—H9B	109.0	C20—C21—H21B	108.6
H9A—C9—H9B	107.8	H21A—C21—H21B	107.6
O1—C10—O2	123.54 (11)	O4—C22—O5	123.84 (12)
O1—C10—C9	123.69 (11)	O4—C22—C21	124.00 (12)
O2—C10—C9	112.77 (11)	O5—C22—C21	112.16 (11)
O3—C11—C8	119.59 (12)	O6—C23—C20	119.67 (13)
O3—C11—C12	120.27 (12)	O6—C23—C24	120.19 (13)
C8—C11—C12	120.14 (11)	C20—C23—C24	120.14 (12)
C11—C12—H12A	109.5	C23—C24—H24A	109.5
C11—C12—H12B	109.5	C23—C24—H24B	109.5
H12A—C12—H12B	109.5	H24A—C24—H24B	109.5
C11—C12—H12C	109.5	C23—C24—H24C	109.5
H12A—C12—H12C	109.5	H24A—C24—H24C	109.5
H12B—C12—H12C	109.5	H24B—C24—H24C	109.5
			
C6—C1—C2—C3	−4.16 (19)	C18—C13—C14—C15	3.5 (2)
C7—C1—C2—C3	174.86 (12)	C19—C13—C14—C15	−176.45 (14)
C1—C2—C3—C4	1.8 (2)	C13—C14—C15—C16	−0.9 (2)
C2—C3—C4—C5	1.4 (2)	C14—C15—C16—C17	−1.6 (3)
C3—C4—C5—C6	−2.2 (2)	C15—C16—C17—C18	1.5 (2)
C4—C5—C6—C1	−0.3 (2)	C16—C17—C18—C13	1.2 (2)
C2—C1—C6—C5	3.4 (2)	C14—C13—C18—C17	−3.6 (2)
C7—C1—C6—C5	−175.55 (13)	C19—C13—C18—C17	176.32 (14)
C6—C1—C7—C8	35.8 (2)	C18—C13—C19—C20	−40.4 (2)
C2—C1—C7—C8	−143.15 (14)	C14—C13—C19—C20	139.55 (15)
C1—C7—C8—C11	176.80 (12)	C13—C19—C20—C23	−176.30 (12)
C1—C7—C8—C9	−0.8 (2)	C13—C19—C20—C21	−2.2 (2)
C7—C8—C9—C10	−98.93 (15)	C19—C20—C21—C22	109.04 (15)
C11—C8—C9—C10	83.34 (14)	C23—C20—C21—C22	−76.51 (14)
C8—C9—C10—O1	−1.06 (18)	C20—C21—C22—O4	−3.04 (19)
C8—C9—C10—O2	179.02 (11)	C20—C21—C22—O5	176.86 (11)
C7—C8—C11—O3	178.72 (13)	C19—C20—C23—O6	171.99 (13)
C9—C8—C11—O3	−3.45 (18)	C21—C20—C23—O6	−2.65 (18)
C7—C8—C11—C12	−1.81 (19)	C19—C20—C23—C24	−8.0 (2)
C9—C8—C11—C12	176.02 (12)	C21—C20—C23—C24	177.38 (12)

### Hydrogen-bond geometry (Å, º)

**Table:**

*D*—H···*A*	*D*—H	H···*A*	*D*···*A*	*D*—H···*A*
O2—H2*A*···O1^i^	0.84 (1)	1.78 (1)	2.6226 (13)	178 (2)
O5—H5*A*···O4^ii^	0.86 (1)	1.74 (1)	2.6000 (13)	176 (2)
C19—H19···O6^iii^	0.95	2.60	3.447 (1)	148

Symmetry codes: (i) −*x*, −*y*+1, −*z*; (ii) −*x*+1, −*y*+1, −*z*+1; (iii) *x*, −*y*+1/2, *z*+1/2.
